# Myeloma Cells with Auer Rod-like Inclusions

**DOI:** 10.5505/tjh.2012.72324

**Published:** 2012-06-15

**Authors:** Abbas Hashim Abdulsalam, Fatin Mohammed Al-yassin

**Affiliations:** 1 Al-Yarmouk Teaching Hospital, Teaching Laboratories Department, Hematology Unit, Baghdad, Iraq; 2 Baghdad Medical City, Teaching Laboratories, Hematology Department, Baghdad, Iraq

**Myeloma cells with Auer-rod like inclusions **

Analysis of bone marrow aspirate obtained from a 47-year-old female that presented with backache showed that she had IgG-κ-type multiple myeloma and renal impairment. FBC showed the following: Hb: 100 g L–1; Hct: 0.34 l/l; WBC: 9.5 x 10^9^ L–1; platelet count: 194 x 10^9^ L^–1^; differential count: normal. 

The presented image showings heavily granulated myeloma cells, some with Auer rod-like inclusions. Auer rod-like inclusions in myeloma are composed of crystallized lysosomal enzyme depositions. They are distinguished from intracytoplasmic immunoglobulin crystals. The factors that predispose to Auer rod-like inclusions in myeloma are unknown; however, in all reported cases paraprotein was of the kappa type. [1]Written informed consent was obtained from the patient.

**Conflict of Interest Statement **

The authors of this paper have no conflicts of interest,including specific financial interests, relationships, and/or affiliations relevant to the subject matter or materials included.

## Figures and Tables

**Figure 1:**
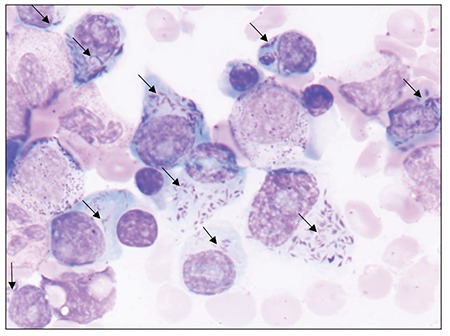
Heavily granulated myeloma cells, some with Auerrod-like granules.
